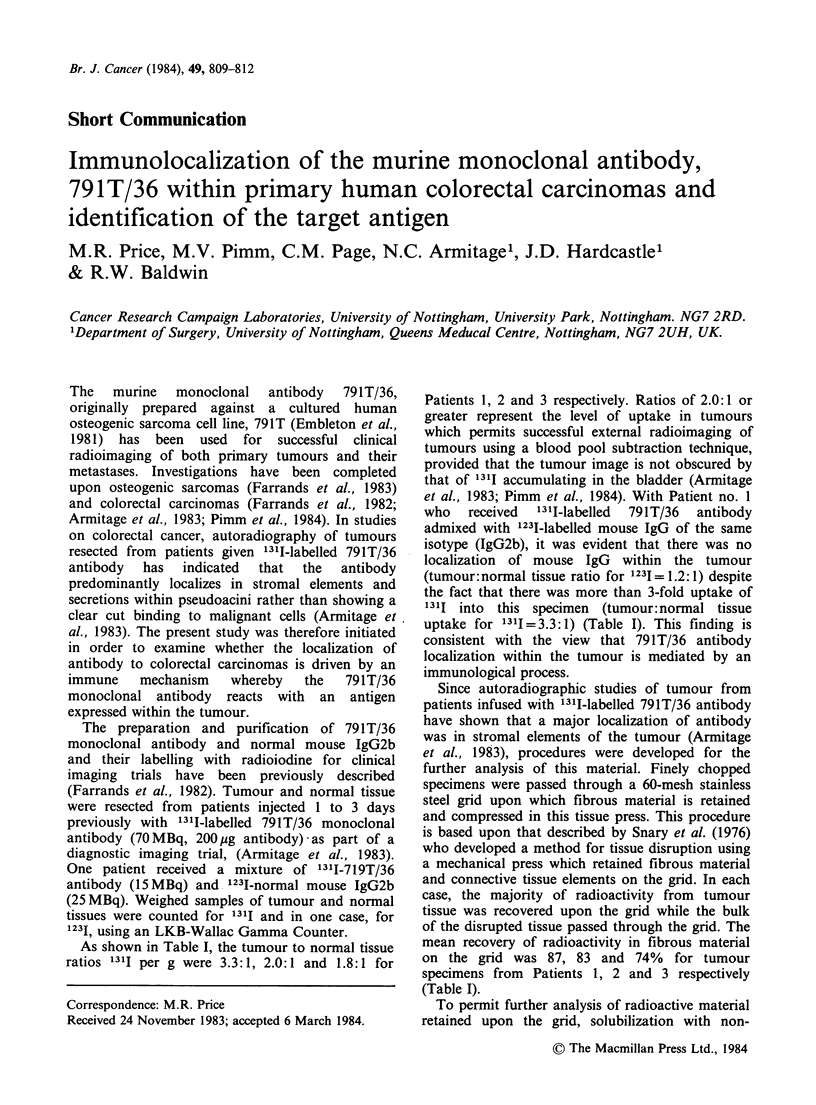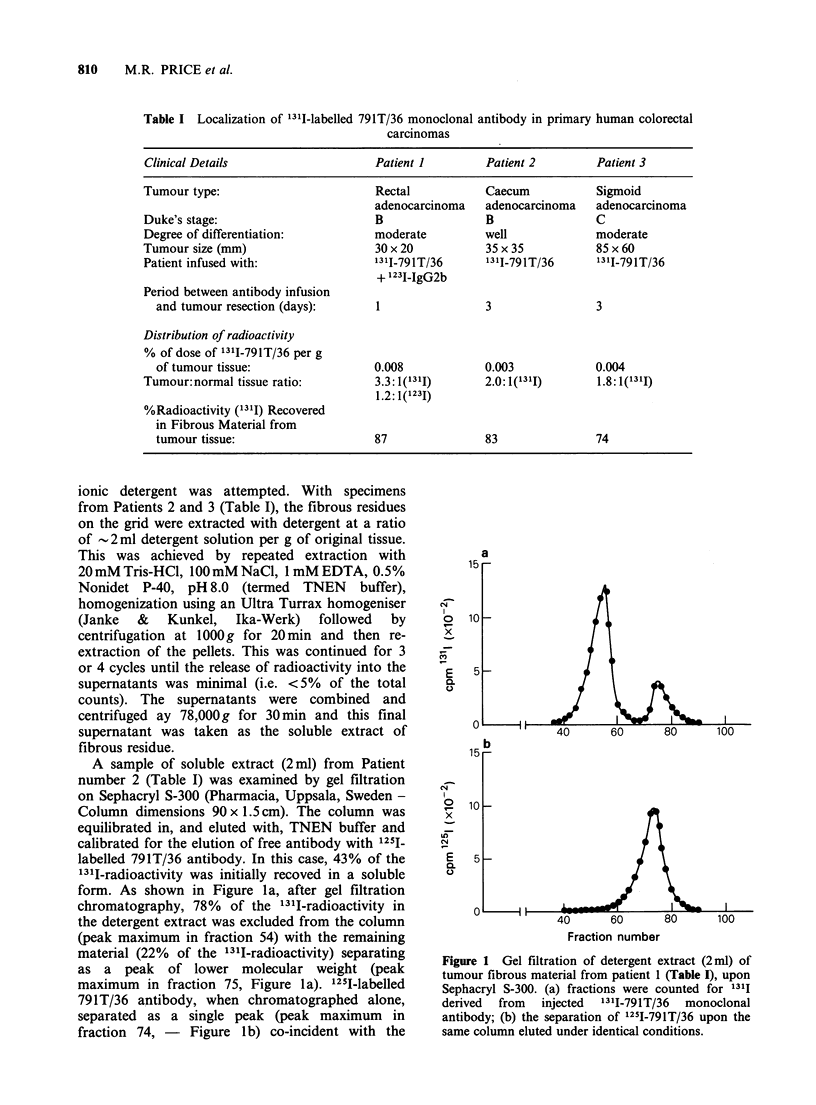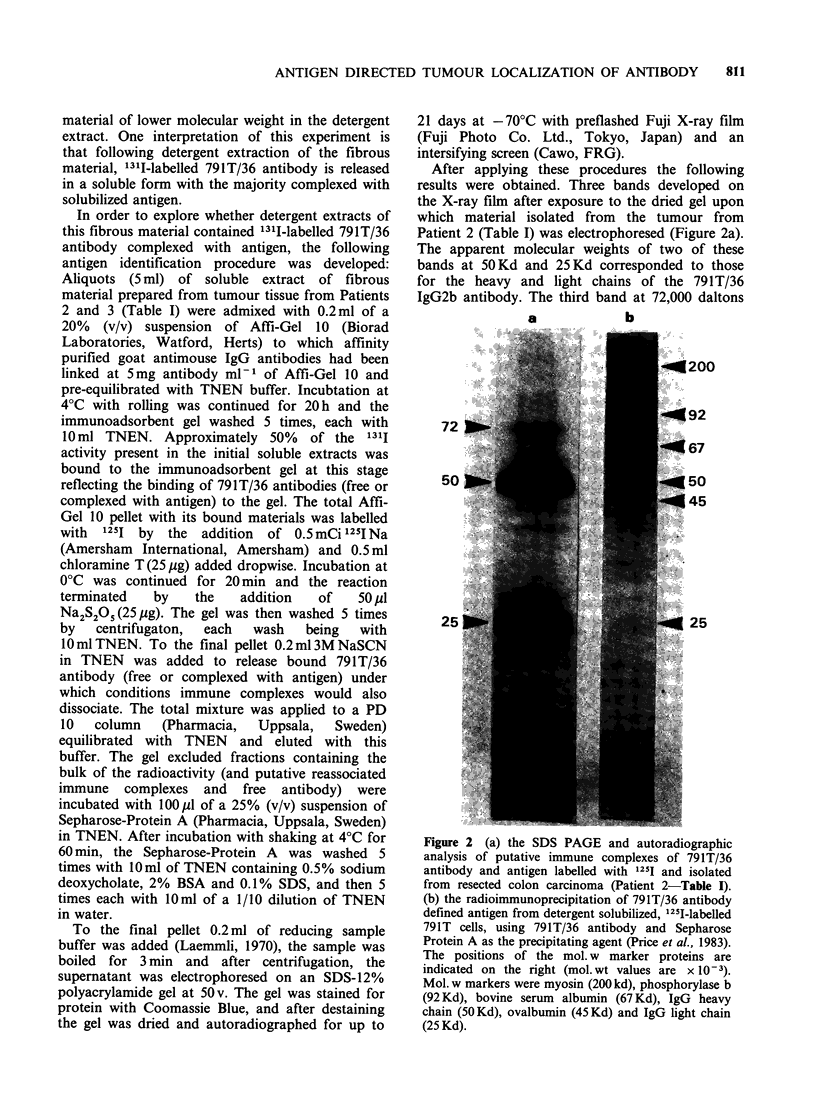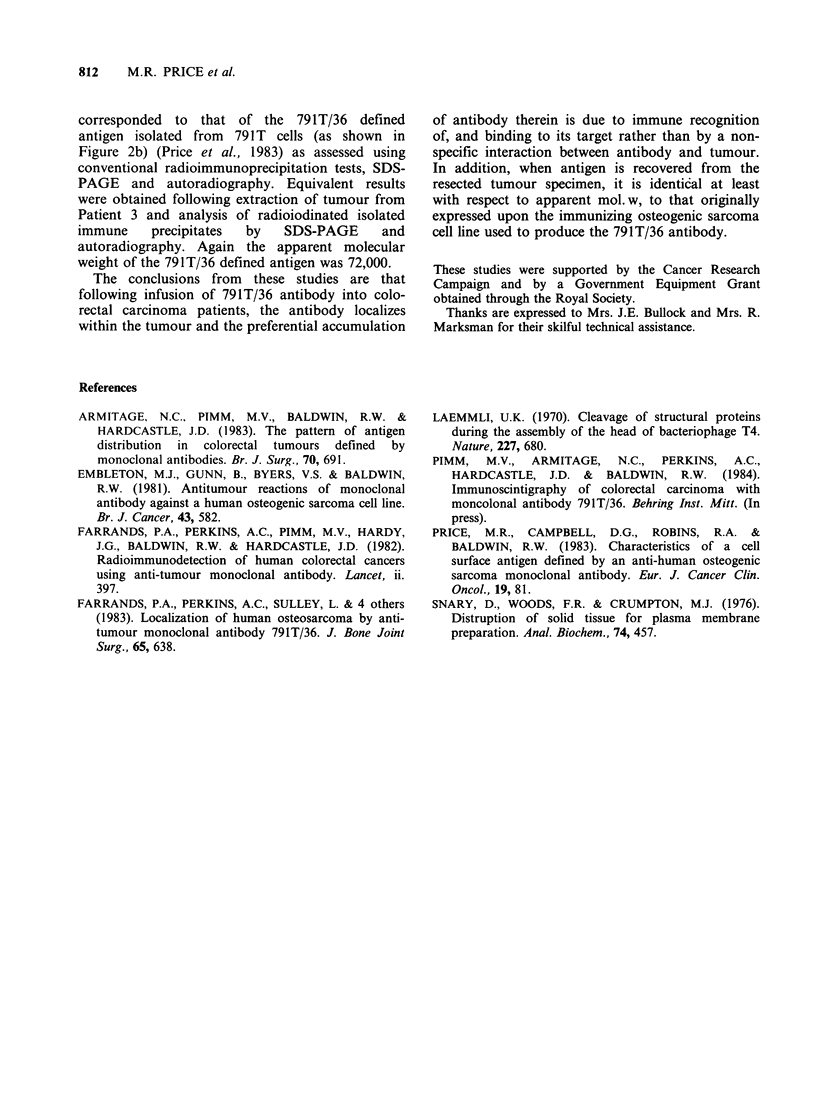# Immunolocalization of the murine monoclonal antibody, 791T/36 within primary human colorectal carcinomas and identification of the target antigen.

**DOI:** 10.1038/bjc.1984.126

**Published:** 1984-06

**Authors:** M. R. Price, M. V. Pimm, C. M. Page, N. C. Armitage, J. D. Hardcastle, R. W. Baldwin

## Abstract

**Images:**


Br. J. Cancer (1984), 49, 809–812

**Short Communication**

### Immunolocalization of the murine monoclonal antibody, 791T/36 within primary human colorectal carcinomas and identification of the target antigen

M.R. Price, M.V. Pimm, C.M. Page, N.C. Armitage[1], J.D. Hardcastle[1] & R.W. Baldwin

*Cancer Research Campaign Laboratories, University of Nottingham, University Park, Nottingham. NG7 2RD.*
[1]*Department of Surgery, University of Nottingham, Queens Meducal Centre, Nottingham, NG7 2UH, UK.*

The murine monoclonal antibody 791T/36, originally prepared against a cultured human osteogenic sarcoma cell line, 791T (Embleton *et al.*, 1981) has been used for successful clinical radioimaging of both primary tumours and their metastases. Investigations have been completed upon osteogenic sarcomas (Farrands *et al.*, 1983) and colorectal carcinomas (Farrands *et al.*, 1982; Armitage *et al.*, 1983; Pimm *et al.*, 1984). In studies on colorectal cancer, autoradiography of tumours resected from patients given $^{131}$I-labelled 791T/36 antibody has indicated that the antibody predominantly localizes in stromal elements and secretions within pseudoacini rather than showing a clear cut binding to malignant cells (Armitage *et al.*, 1983). The present study was therefore initiated in order to examine whether the localization of antibody to colorectal carcinomas is driven by an immune mechanism whereby the 791T/36 monoclonal antibody reacts with an antigen expressed within the tumour.

The preparation and purification of 791T/36 monoclonal antibody and normal mouse IgG2b and their labelling with radioiodine for clinical imaging trials have been previously described (Farrands *et al.*, 1982). Tumour and normal tissue were resected from patients injected 1 to 3 days previously with $^{131}$I-labelled 791T/36 monoclonal antibody (70 MBq, 200 $\mu$g antibody) as part of a diagnostic imaging trial, (Armitage *et al.*, 1983). One patient received a mixture of $^{131}$I-719T/36 antibody (15 MBq) and $^{123}$I-normal mouse IgG2b (25 MBq). Weighed samples of tumour and normal tissues were counted for $^{131}$I and in one case, for $^{123}$I, using an LKB-Wallac Gamma Counter.

As shown in Table I, the tumour to normal tissue ratios $^{131}$I per g were 3.3:1, 2.0:1 and 1.8:1 for Patients 1, 2 and 3 respectively. Ratios of 2.0:1 or greater represent the level of uptake in tumours which permits successful external radioimaging of tumours using a blood pool subtraction technique, provided that the tumour image is not obscured by that of $^{131}$I accumulating in the bladder (Armitage *et al.*, 1983; Pimm *et al.*, 1984). With Patient no. 1 who received $^{131}$I-labelled 791T/36 antibody admixed with $^{123}$I-labelled mouse IgG of the same isotype (IgG2b), it was evident that there was no localization of mouse IgG within the tumour (tumour:normal tissue ratio for $^{123}$I = 1.2:1) despite the fact that there was more than 3-fold uptake of $^{131}$I into this specimen (tumour:normal tissue uptake for $^{131}$I = 3.3:1) (Table I). This finding is consistent with the view that 791T/36 antibody localization within the tumour is mediated by an immunological process.

Since autoradiographic studies of tumour from patients infused with $^{131}$I-labelled 791T/36 antibody have shown that a major localization of antibody was in stromal elements of the tumour (Armitage *et al.*, 1983), procedures were developed for the further analysis of this material. Finely chopped specimens were passed through a 60-mesh stainless steel grid upon which fibrous material is retained and compressed in this tissue press. This procedure is based upon that described by Snary *et al.* (1976) who developed a method for tissue disruption using a mechanical press which retained fibrous material and connective tissue elements on the grid. In each case, the majority of radioactivity from tumour tissue was recovered upon the grid while the bulk of the disrupted tissue passed through the grid. The mean recovery of radioactivity in fibrous material on the grid was 87, 83 and 74% for tumour specimens from Patients 1, 2 and 3 respectively (Table I).

To permit further analysis of radioactive material retained upon the grid, solubilization with non-

Correspondence: M.R. Price
Received 24 November 1983; accepted 6 March 1984.

© The Macmillan Press Ltd., 1984

**Table I** Localization of $^{131}$I-labelled 791T/36 monoclonal antibody in primary human colorectal carcinomas

| *Clinical Details* | *Patient 1* | *Patient 2* | *Patient 3* |
|---|---|---|---|
| Tumour type: | Rectal adenocarcinoma | Caecum adenocarcinoma | Sigmoid adenocarcinoma |
| Duke's stage: | B | B | C |
| Degree of differentiation: | moderate | well | moderate |
| Tumour size (mm) | 30 x 20 | 35 x 35 | 85 x 60 |
| Patient infused with: | $^{131}$I-791T/36 + $^{123}$I-IgG2b | $^{131}$I-791T/36 | $^{131}$I-791T/36 |
| Period between antibody infusion and tumour resection (days): | 1 | 3 | 3 |
| *Distribution of radioactivity* | | | |
| % of dose of $^{131}$I-791T/36 per g of tumour tissue: | 0.008 | 0.003 | 0.004 |
| Tumour:normal tissue ratio: | 3.3:1($^{131}$I) 1.2:1($^{123}$I) | 2.0:1($^{131}$I) | 1.8:1($^{131}$I) |
| %Radioactivity ($^{131}$I) Recovered in Fibrous Material from tumour tissue: | 87 | 83 | 74 |

ionic detergent was attempted. With specimens from Patients 2 and 3 (Table I), the fibrous residues on the grid were extracted with detergent at a ratio of ~2 ml detergent solution per g of original tissue. This was achieved by repeated extraction with 20 mM Tris-HCl, 100 mM NaCl, 1 mM EDTA, 0.5% Nonidet P-40, pH 8.0 (termed TNEN buffer), homogenization using an Ultra Turrax homogeniser (Janke & Kunkel, Ika-Werk) followed by centrifugation at 1000 *g* for 20 min and then re-extraction of the pellets. This was continued for 3 or 4 cycles until the release of radioactivity into the supernatants was minimal (i.e. <5% of the total counts). The supernatants were combined and centrifuged ay 78,000 *g* for 30 min and this final supernatant was taken as the soluble extract of fibrous residue.

A sample of soluble extract (2 ml) from Patient number 2 (Table I) was examined by gel filtration on Sephacryl S-300 (Pharmacia, Uppsala, Sweden – Column dimensions 90 x 1.5 cm). The column was equilibrated in, and eluted with, TNEN buffer and calibrated for the elution of free antibody with $^{125}$I-labelled 791T/36 antibody. In this case, 43% of the $^{131}$I-radioactivity was initially recoved in a soluble form. As shown in Figure 1a, after gel filtration chromatography, 78% of the $^{131}$I-radioactivity in the detergent extract was excluded from the column (peak maximum in fraction 54) with the remaining material (22% of the $^{131}$I-radioactivity) separating as a peak of lower molecular weight (peak maximum in fraction 75, Figure 1a). $^{125}$I-labelled 791T/36 antibody, when chromatographed alone, separated as a single peak (peak maximum in fraction 74, — Figure 1b) co-incident with the

**Figure 1** Gel filtration of detergent extract (2 ml) of tumour fibrous material from patient 1 (**Table I**), upon Sephacryl S-300. (a) fractions were counted for $^{131}$I derived from injected $^{131}$I-791T/36 monoclonal antibody; (b) the separation of $^{125}$I-791T/36 upon the same column eluted under identical conditions.

material of lower molecular weight in the detergent extract. One interpretation of this experiment is that following detergent extraction of the fibrous material, $^{131}$I-labelled 791T/36 antibody is released in a soluble form with the majority complexed with solubilized antigen.

In order to explore whether detergent extracts of this fibrous material contained $^{131}$I-labelled 791T/36 antibody complexed with antigen, the following antigen identification procedure was developed: Aliquots (5 ml) of soluble extract of fibrous material prepared from tumour tissue from Patients 2 and 3 (Table I) were admixed with 0.2 ml of a 20% (v/v) suspension of Affi-Gel 10 (Biorad Laboratories, Watford, Herts) to which affinity purified goat antimouse IgG antibodies had been linked at 5 mg antibody ml$^{-1}$ of Affi-Gel 10 and pre-equilibrated with TNEN buffer. Incubtation at 4°C with rolling was continued for 20 h and the immunoadsorbent gel washed 5 times, each with 10 ml TNEN. Approximately 50% of the $^{131}$I activity present in the initial soluble extracts was bound to the immunoadsorbent gel at this stage reflecting the binding of 791T/36 antibodies (free or complexed with antigen) to the gel. The total Affi-Gel 10 pellet with its bound materials was labelled with $^{125}$I by the addition of 0.5 mCi $^{125}$I Na (Amersham International, Amersham) and 0.5 ml chloramine T (25 $\mu$g) added dropwise. Incubation at 0°C was continued for 20 min and the reaction terminated by the addition of 50 $\mu$l $Na_2S_2O_5$ (25 $\mu$g). The gel was then washed 5 times by centrifugaton, each wash being with 10 ml TNEN. To the final pellet 0.2 ml 3M NaSCN in TNEN was added to release bound 791T/36 antibody (free or complexed with antigen) under which conditions immune complexes would also dissociate. The total mixture was applied to a PD 10 column (Pharmacia, Uppsala, Sweden) equilibrated with TNEN and eluted with this buffer. The gel excluded fractions containing the bulk of the radioactivity (and putative reassociated immune complexes and free antibody) were incubated with 100 $\mu$l of a 25% (v/v) suspension of Sepharose-Protein A (Pharmacia, Uppsala, Sweden) in TNEN. After incubation with shaking at 4°C for 60 min, the Sepharose-Protein A was washed 5 times with 10 ml of TNEN containing 0.5% sodium deoxycholate, 2% BSA and 0.1% SDS, and then 5 times each with 10 ml of a 1/10 dilution of TNEN in water.

To the final pellet 0.2 ml of reducing sample buffer was added (Laemmli, 1970), the sample was boiled for 3 min and after centrifugation, the supernatant was electrophoresed on an SDS-12% polyacrylamide gel at 50 v. The gel was stained for protein with Coomassie Blue, and after destaining the gel was dried and autoradiographed for up to 21 days at $-70$°C with preflashed Fuji X-ray film (Fuji Photo Co. Ltd., Tokyo, Japan) and an intersifying screen (Cawo, FRG).

After applying these procedures the following results were obtained. Three bands developed on the X-ray film after exposure to the dried gel upon which material isolated from the tumour from Patient 2 (Table I) was electrophoresed (Figure 2a). The apparent molecular weights of two of these bands at 50 Kd and 25 Kd corresponded to those for the heavy and light chains of the 791T/36 IgG2b antibody. The third band at 72,000 daltons

**Figure 2** (a) the SDS PAGE and autoradiographic analysis of putative immune complexes of 791T/36 antibody and antigen labelled with $^{125}$I and isolated from resected colon carcinoma (Patient 2—**Table I**). (b) the radioimmunoprecipitation of 791T/36 antibody defined antigen from detergent solubilized, $^{125}$I-labelled 791T cells, using 791T/36 antibody and Sepharose Protein A as the precipitating agent (Price *et al.*, 1983). The positions of the mol. w marker proteins are indicated on the right (mol. wt values are $\times 10^{-3}$). Mol. w markers were myosin (200 kd), phosphorylase b (92 Kd), bovine serum albumin (67 Kd), IgG heavy chain (50 Kd), ovalbumin (45 Kd) and IgG light chain (25 Kd).

corresponded to that of the 791T/36 defined antigen isolated from 791T cells (as shown in Figure 2b) (Price *et al.*, 1983) as assessed using conventional radioimmunoprecipitation tests, SDS-PAGE and autoradiography. Equivalent results were obtained following extraction of tumour from Patient 3 and analysis of radioiodinated isolated immune precipitates by SDS-PAGE and autoradiography. Again the apparent molecular weight of the 791T/36 defined antigen was 72,000.

The conclusions from these studies are that following infusion of 791T/36 antibody into colorectal carcinoma patients, the antibody localizes within the tumour and the preferential accumulation of antibody therein is due to immune recognition of, and binding to its target rather than by a non-specific interaction between antibody and tumour. In addition, when antigen is recovered from the resected tumour specimen, it is identical at least with respect to apparent mol. w, to that originally expressed upon the immunizing osteogenic sarcoma cell line used to produce the 791T/36 antibody.

These studies were supported by the Cancer Research Campaign and by a Government Equipment Grant obtained through the Royal Society.

Thanks are expressed to Mrs. J.E. Bullock and Mrs. R. Marksman for their skilful technical assistance.

### References

ARMITAGE, N.C., PIMM, M.V., BALDWIN, R.W. & HARDCASTLE, J.D. (1983). The pattern of antigen distribution in colorectal tumours defined by monoclonal antibodies. *Br. J. Surg.*, **70**, 691.

EMBLETON, M.J., GUNN, B., BYERS, V.S. & BALDWIN, R.W. (1981). Antitumour reactions of monoclonal antibody against a human osteogenic sarcoma cell line. *Br. J. Cancer*, **43**, 582.

FARRANDS, P.A., PERKINS, A.C., PIMM, M.V., HARDY, J.G., BALDWIN, R.W. & HARDCASTLE, J.D. (1982). Radioimmunodetection of human colorectal cancers using anti-tumour monoclonal antibody. *Lancet*, ii. 397.

FARRANDS, P.A., PERKINS, A.C., SULLEY, L. & 4 others (1983). Localization of human osteosarcoma by anti-tumour monoclonal antibody 791T/36. *J. Bone Joint Surg.*, **65**, 638.

LAEMMLI, U.K. (1970). Cleavage of structural proteins during the assembly of the head of bacteriophage T4. *Nature*, **227**, 680.

PIMM, M.V., ARMITAGE, N.C., PERKINS, A.C., HARDCASTLE, J.D. & BALDWIN, R.W. (1984). Immunoscintigraphy of colorectal carcinoma with moncolonal antibody 791T/36. *Behring Inst. Mitt.* (In press).

PRICE, M.R., CAMPBELL, D.G., ROBINS, R.A. & BALDWIN, R.W. (1983). Characteristics of a cell surface antigen defined by an anti-human osteogenic sarcoma monoclonal antibody. *Eur. J. Cancer Clin. Oncol.*, **19**, 81.

SNARY, D., WOODS, F.R. & CRUMPTON, M.J. (1976). Distruption of solid tissue for plasma membrane preparation. *Anal. Biochem.*, **74**, 457.